# Clinical practice and postoperative rehabilitation after knee arthroscopy vary according to surgeons’ expertise: a survey among polish arthroscopy society members

**DOI:** 10.1186/s12891-020-03649-9

**Published:** 2020-09-23

**Authors:** Paweł Bąkowski, Kamilla Bąkowska-Żywicka, Tomasz Piontek

**Affiliations:** 1 Department of Orthopedic Surgery, Rehasport Clinic, Górecka Street 30, 60201 Poznan, Poland; 2 Institute of Bioorganic Chemistry Polish Academy of Sciences, Noskowkiego 12/14, 61-704 Poznań, Poland; 3 Department of Spine Disorders and Pediatric Orthopedics, University of Medical Sciences Poznan, Poznan, Poland

**Keywords:** Knee arthroscopy, Meniscus repair, Survey, Surgical expertise, Rehabilitation, Return to sport

## Abstract

**Background:**

Meniscus repair is a challenging task in knee arthroscopy. Currently, there are a variety of arthroscopic methods available for meniscus repair. The purpose of this study was to determine a consensus in meniscus tear treatment in the environment of Polish orthopaedists.

**Methods:**

A total of 205 registered orthopaedic surgeons participated in the surveys. The survey consisted of 35 questions regarding general arthroscopy and postoperative management, including physicians’ level of expertise, anaesthesia, postoperative treatment, rehabilitation and procedures performed. Comparisons were made between knee arthroscopy experts (> 100 arthroscopies performed per year) and non-experts (≤ 100 cases) on aspects of patient care.

**Results:**

The most important finding of this study was the agreement among almost all aspects of the knee arthroscopy approach. Consensus among Polish surgeons was noticed in choosing regional anaesthesia for knee arthroscopy, the lack of need for knee braces and knee medications, the of use of LMW heparin for thromboprophylaxis, 1–2 days of hospitalization, the recommendation of rehabilitation and the use of magnetic resonance as a diagnostic test for meniscus damage. Surgical expertise was significantly associated with the performance of meniscus suture procedures (*p* = 0.009). Experts recommended starting rehabilitation on the day of surgery (*p* = 0.007) and were more likely to use objective physical tests (*p* = 0.003). Non-expert surgeons recommended a longer period from meniscus suture to full-range knee motion (*p* = 0.001) and admitted that patient age does matter for meniscus repair qualification (*p* = 0.002).

**Conclusions:**

There is consensus among almost all issues of meniscus tear treatment in the environment of Polish orthopaedists; however, the issues of rehabilitation and the use of advanced meniscus repair techniques are associated with surgical expertise.

## Background

Meniscus injuries represent a common ailment of knee damage [1]. Due to the hypovascularity and hypocellularity of the meniscus, meniscus tears do not heal spontaneously [2–4], and prolonged untreated damage may lead to the development of osteoarthritis [5–7]. Current orthopaedic practice aims to preserve meniscal integrity and restore function [8–11]. A variety of arthroscopic methods of meniscus repair are used, including different suturing techniques, which are often modified to increase effectiveness [12–15]. Therefore, studies aimed at the analysis of existing meniscus repair methods are very important. Recently, the European Society for Sports Traumatology, Knee Surgery and Arthroscopy (ESSKA) consensus provided recommendations for the treatment of meniscus tears based on both scientific evidence and the clinical experience of expert knee surgeons [16, 17]. Other studies have shown that a surgeon’s level of expertise significantly affects clinical outcomes in patients undergoing knee arthroscopy [18, 19]. Therefore, the main goal of the study was to determine a consensus in meniscus tear treatment in the environment of Polish orthopaedists. We hypothesized that clinical practice would differ among the Polish Arthroscopy Society community members according to the level of surgical expertise.

## Methods

For this study, a questionnaire was presented to 205 orthopaedists with various levels of clinical expertise in arthroscopy during the Polish Arthroscopy Society Congress, which was held on 24–26 October 2019 in Katowice, Poland. The questionnaire consisted of six sections with a total of 35 questions regarding knee arthroscopy and postoperative management. The six sections of the questionnaire were as follows: (A) the physician’s level of expertise (4 questions), (B) anaesthesia during arthroscopy (1 question), (C) postoperative treatment (3 questions), (D) hospital stay (3 questions), (E) rehabilitation protocols (11 questions) and (F) procedures in arthroscopy (13 questions). The complete survey is available in Additional file 1.

A pilot survey was conducted before the meeting. The questionnaire was distributed to 10 orthopaedic surgeons and a biostatistician to ensure that it was scientifically sound and that the question stems were easy to understand. We defined experts as any participating orthopaedist who had performed > 100 knee arthroscopies per year. Orthopaedists who had performed 100 or fewer knee arthroscopies per year were classified as non-experts for this study.

### Statistical analysis

Statistics were conducted using Prism8 software (GraphPad Software, San Diego, CA). Power analysis was conducted to identify the minimum number of participants required in each group to detect statistical significance. The sample size calculation showed that with a power of 80% (2-sided testing at a significance level of 0.05), a sample size of 43 participants was needed. To test proportional differences in categorical variables, a Chi-square test was performed. Fisher’s exact test was used when cells contained less than five subjects. Statistical significance was determined at *p* < 0.05.

## Results

A total of 205 participants received questionnaires. All survey forms were used for the analysis. Table 1 presents the educational background of the participating surgeons in the field of knee arthroscopy. Fifty-five orthopaedists (28%) performed more than 100 knee arthroscopies per year independently and were classified as experts for this study. The remaining 150 orthopaedists (72%) performed up to 100 knee arthroscopies per year and were therefore classified as non-experts.

**Table 1:** Physician’s level of education and experience

Parameter:	Parameter value:	Surgeons (*n* = 205)
Participation in the knee arthroscopy during residency or specialization	0	8 (4%)
	1–30	28 (14%)
	> 30	169 (82%)
The independent knee arthroscopies performed per year	0–50	98 (47%)
	50–100	52 (25%)
	> 100	55 (28%)
The independent knee arthroscopies performed during career	0–500	127 (62%)
	> 500	78 (38%)
The joints currently subjected to arthroscopy procedures	shoulder	83 (41%)
	elbow	28 (14%)
	wrist	9 (4%)
	spine	1 (0.5%)
	hip	41 (20%)
	knee	200 (98%)
	ankle	82 (40%)

The comparison of the clinical practice between expert and non-expert surgeons who performed knee arthroscopy is shown in Table 2. Regional anaesthesia (spinal/epidural) was favoured by 172 (84%) orthopaedists (48 experts and 124 non-experts). Only 27 (13%) orthopaedists (4 experts and 23 non-experts) recommended the use of an orthosis to their patients immediately after knee arthroscopy. Only 22 (10%) orthopaedists (8 experts and 10 non-experts) used knee medications in the first 24 h after arthroscopy. The most commonly reported pain medications were local anaesthetic drugs belonging to the amino amide group (8 surgeons) or hyaluronic acid (7 surgeons). Experts and non-experts answered almost equally when asked about anti-thrombotic prophylaxis administered to patients. Low-molecular-weight heparin (LMH) was recommended by 181 (88%) surgeons (51 experts and 130 non-experts). Both knee arthroscopy experts (85%) and non-experts (75%) recommended 1 day of hospitalization after non-reconstructive arthroscopy. One or 2 days of hospitalization were most frequently recommended after reconstructive arthroscopy.

**Table 2:** Comparison of the clinical practice between expert and non-expert surgeons who perform knee arthroscopy

Parameter:	All (*n* = 205)	Experts (*n* = 55)	Non-experts (*n* = 150)	*p* value
Use of regional anesthesia	172 (84%)	48 (87%)	124 (83%)	n.s.
Recommend orthosis	27 (13%)	4 (7%)	23 (15%)	n.s.
Use of a knee drain	189 (92%)	50 (91%)	139 (72%)	0.012
Use of knee medications	22 (10%)	8 (15%)	10 (7%)	n.s.
Recommend LMW as thromboprophylaxis	181 (88%)	51 (94%)	130 (87%)	n.s.
One day hospitalization after non-reconstructive arthroscopy	160 (77%)	47 (85%)	113 (75%)	n.s.
One day of hospitalization after reconstructive arthroscopy	81 (39%)	29 (52%)	52 (35%)	n.s.
Two days of hospitalization after reconstructive arthroscopy	90 (44%)	17 (31%)	73 (49%)	n.s.

*p* value is presented to establish statistical significance between expert and non-expert treatment. *n.s.* not significant*, LMW heparin* low molecular weight heparin

The comparison of the rehabilitation recommendations is shown in Table 3. A total of 203 (99%) surgeons (55 experts and 148 non-experts) reported that they always discussed the importance of rehabilitation with the patients. A total of 135 (64%) surgeons always recommend rehabilitation (excluding physical therapy), and 43 (21%) mostly recommend rehabilitation. There was a statistically significant difference (*p* = 0.032) when surgeons were asked about their patients’ compliance with the rehabilitation recommendations. A total of 22% of experts and 14% of non-experts admitted that their patients mostly followed the rehabilitation recommendations. A total of 124 (60%) surgeons recommended beginning rehabilitation within 1 day after surgery. Knee arthroscopy experts more frequently recommended beginning rehabilitation on the day of surgery (14 of 55 experts, 25%, *p* = 0.007). A standardized rehabilitation protocol was recommended by 84 (42%) surgeons. A total of 176 (86%) surgeons reported that the rehabilitation protocol was dependent on the procedure performed. A total of 189 (92%) surgeons reported that the physical therapist was the key person responsible for patient rehabilitation. Cryotherapy was recommended by 77% of orthopaedists (42 experts and 113 non-experts) and physical therapy by 65%. Within this group, laser therapy and magnetotherapy were most frequently used.

**Table 3:** Comparison of post-surgical rehabilitation recommendations between expert and non-expert surgeons who perform knee arthroscopy

Parameter:	All (*n* = 205)	Experts (*n* = 55)	Non-experts (*n* = 150)	*p* value
Talk about the need for rehabilitation	203 (99%)	55 (100%)	148 (99%)	n.s.
Always recommend rehabilitation	135 (64%)	41 (75%)	94 (63%)	n.s.
Mostly recommend rehabilitation	43 (21%)	8 (14%)	35 (23%)	n.s.
Patients mostly follow the rehabilitation recommendations	28 (14%)	12 (22%)	16 (14%)	0.032
Patients sometimes follow the rehabilitation recommendations	122 (60%)	30 (55%)	92 (61%)	n.s.
Beginning of rehabilitation on the day of surgery	22 (11%)	14 (25%)	8 (5%)	0.007
Beginning of rehabilitation one day after the surgery	124 (60%)	33 (60%)	91 (61%)	
Recommend standardized rehabilitation	84 (42%)	21 (38%)	63 (43%)	n.s.
Dependence of rehabilitation program on performed procedure	176 (86%)	47 (85%)	129 (63%)	n.s.
Recommend rehabilitation with a physiotherapist	189 (92%)	49 (89%)	140 (93%)	n.s.
Recommend cryotherapy	158 (77%)	45 (82%)	113 (75%)	n.s.
Recommend physical therapy	133 (65%)	29 (53%)	104 (69%)	0.04
Recommend lasertherapy	69 (34%)	16 (29%)	53 (35%)	n.s.
Recommend magnetotherapy	71 (34%)	7 (13%)	51 (34%)	n.s.
Recommend ultrasounds	58 (28%)	18 (32%)	53 (35%)	n.s.
Recommend ionophoresis	39 (19%)	8 (14%)	31 (20%)	n.s.
Recommend TENS	38 (19%)	7 (13%)	31 (20%)	n.s.

*p* value is presented to establish statistical significance between expert and non-expert treatment. *n.s.* not significant

Table 4 shows the factors considered when recommending return to sport activity after knee arthroscopy. In most cases, either the surgeon alone or the surgeon together with a physical therapist were responsible for the decision of whether a patient was ready to return to sport. The most important factor in the decision process was the functional state of the patient (93% of experts and 74% non-experts, *p* = 0.002). Objective measurements were used to aid in the decision of whether to return to sport by 159 (78%) surgeons (50 experts and 109 non-experts, *p* = 0.003). Among them, functional tests were preferred by experts (*p* = 0.006).

**Table 4:** Comparison of return to sport recommendations between expert and non-expert surgeons who perform knee arthroscopy

Parameter:	All (*n* = 205)	Experts (*n* = 55)	Non-experts (*n* = 150)	*p* value
Decision by surgeon	77 (37%)	23 (45%)	54 (36%)	n.s.
Decision by surgeon and physical therapist	87 (42%)	30 (54%)	57 (38%)	n.s.
Functional state as a decisive criterion	162 (80%)	51 (93%)	111 (74%)	0.002
Lack of discomfort as a decisive criterion	86 (42%)	19 (34%)	67 (45%)	n.s.
Time since arthroscopy as a decisive criterion	72 (35%)	24 (44%)	48 (32%)	n.s.
Correct image in examination as a decisive criterion	39 (19%)	12 (22%)	27 (18%)	n.s.
Use of objective physical tests	159 (78%)	50 (91%)	109 (66%)	0.003
Use of functional tests	119 (58%)	44 (80%)	75 (50%)	0.006
Use of dynamometer	58 (28%)	22 (40%)	36 (24%)	n.s.
Use of subjective surveys	35 (17%)	11 (20%)	24 (16%)	n.s.

*p* value is presented to establish statistical significance between expert and non-expert treatment. *n.s.* not significant

**Fig. 1:**
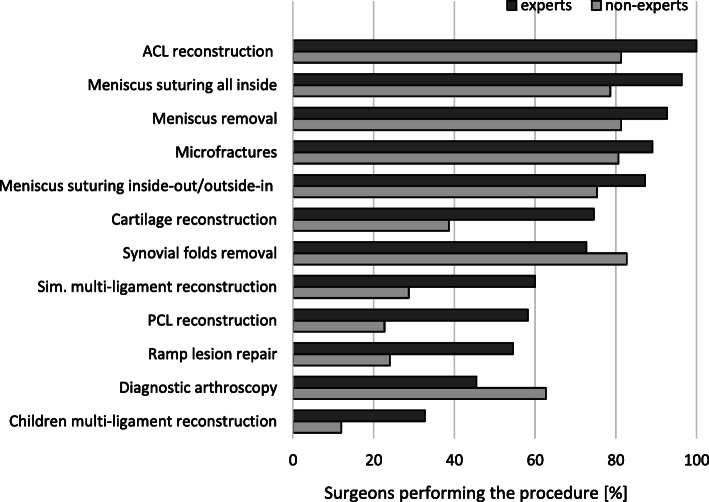
Procedures performed by the participating surgeons

**Fig. 2:**
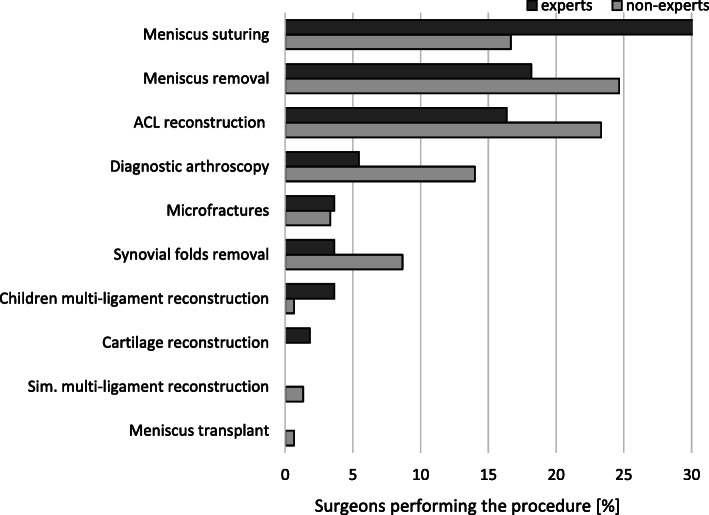
Procedures most frequently performed by the participating surgeons

**Fig. 3:**
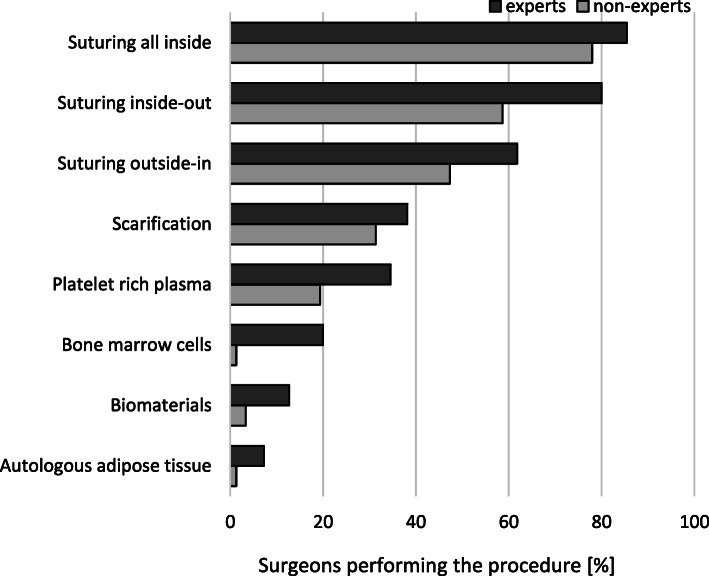
Meniscus repair methods performed by the participating surgeons

**Fig. 4:**
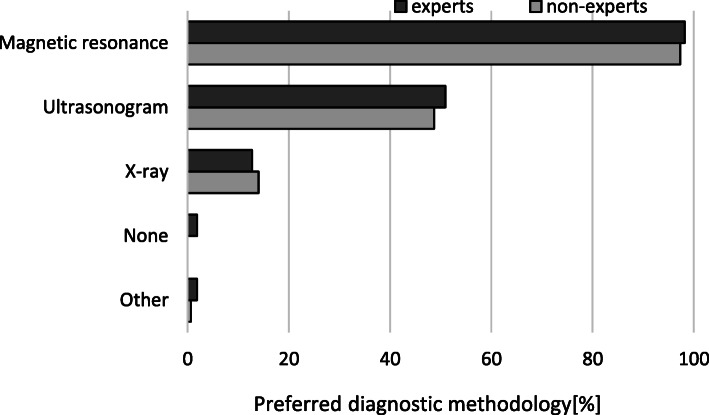
Diagnostic methods used by the participating surgeons

The comparison of the knee arthroscopic procedures performed by expert and non-expert surgeons is shown in Table 5. Both experts and non-experts used a broad spectrum of arthroscopic procedures (Fig. 1). Non-experts had significantly less experience with meniscus suturing (*p* = 0.005) and more experience with meniscus removal (*p* = 0.009) (Fig. 2). Experts used significantly more meniscus repair techniques than non-experts (Fig. 3). The diagnostic tests used by experts and non-experts were similar (n.s.), with magnetic resonance being the preferred diagnostic method (Fig. 4).

**Table 5:** Comparison of knee arthroscopic procedures performed by expert and non-expert surgeons

Parameter:	All (*n* = 205)	Experts (*n* = 55)	Non-experts (*n* = 150)	*p* value
KNEE ARTHROSCOPY PROCEDURES USED:				
ACL reconstruction	177 (86%)	55 (100%)	122 (81%)	0.003
Meniscus removal	173 (84%)	51 (93%)	122 (81%)	n.s.
Meniscus suturing all inside	171 (83%)	53 (96%)	118 (79%)	n.s.
Synovial folds removal	164 (80%)	40 (73%)	124 (83%)	n.s.
Meniscus suturing inside-out/outside-in	161 (79%)	48 (87%)	113 (75%)	n.s.
Microfractures	170 (83%)	49 (89%)	121 (81%)	n.s.
Diagnostic arthroscopy	119 (58%)	25 (45%)	94 (63%)	< 0.001
Cartilage reconstruction	99 (48%)	41 (75%)	58 (39%)	< 0.001
Simultaneous multi-ligament reconstruction	76 (37%)	33 (60%)	43 (29%)	< 0.001
PCL reconstruction	66 (32%)	32 (58%)	34 (23%)	< 0.001
Ramp lesion repair	66 (32%)	30 (55%)	36 (24%)	0.004
Pediatric multi-ligament reconstruction	36 (18%)	18 (33%)	18 (12%)	0.004
Meniscus transplant	32 (16%)	17 (31%)	15 (10%)	< 0.001
KNEE ARTHROSCOPY PROCEDURES USED MOST FREQUENTLY:				
Meniscus removal	47 (23%)	10 (18%)	37 (25%)	0.009
Meniscus suturing	45 (22%)	21 (38%)	25 (17%)	0.005
ACL reconstruction	44 (21%)	9 (16%)	35 (23%)	n.s.
MENISCUS REPAIR METHODS USED				
Suturing all inside	164 (80%)	47 (85%)	117 (78%)	0.009
Suturing inside-out	132 (64%)	44 (80%)	88 (59%)	0.006
Suturing outside-out	105 (51%)	34 (62%)	71 (47%)	0.006
Scarification	68 (33%)	21 (38%)	47 (31%)	n.s.
Platelet rich plasma	48 (23%)	19 (35%)	29 (19%)	0.009
Bone marrow cells	13 (6%)	11 (20%)	2 (1%)	< 0.001
Biomaterials	12 (6%)	7 (13%)	5 (3%)	< 0.001
Autologous adipose tissue	6 (3%)	4 (7%)	2 (1%)	< 0.001
DIAGNOSTIC TESTS USED:				
Magnetic resonance	200 (98%)	54 (98%)	146 (97%)	n.s.
Ultrasonogram	101 (49%)	28 (51%)	73 (49%)	n.s.
X-ray	28 (14%)	7 (13%)	21 (14%)	n.s.

*p* value is presented to establish statistical significance between expert and non-expert treatment. *n.s.* not significant

The comparison of the post-arthroscopic procedures performed by expert and non-expert surgeons is shown in Table 6. Both experts and non-experts recommended similar times of elbow crutch use after meniscus removal (2 weeks) or orthosis after meniscus suturing (6 weeks). However, the answers were different when surgeons were asked about how soon after meniscus suturing they recommended full range of knee motion. Experts recommended 4 weeks, and non-experts recommended 6 weeks (*p* = 0.001).

**Table 6:** Comparison of post-arthroscopic procedures performed by expert and non-expert surgeons

Parameter:	All (*n* = 205)	Experts (*n* = 55)	Non-experts (*n* = 150)	*p* value
Recommend using elbow crutches for 2 weeks	57 (28%)	13 (24%)	54 (36%)	n.s.
Recommend using elbow crutches for 1–6 days	34 (17%)	11 (20%)	23 (15%)	n.s.
Recommend using orthosis for 6 weeks	70 (34%)	19 (35%)	51 (30%)	n.s.
Recommend a full range of knee motion after 6 weeks	79 (39%)	19 (34%)	60 (40%)	0.001
Recommend a full range of knee motion after 4 weeks	48 (24%)	18 (33%)	30 (20%)	0.001

*p* value is presented to establish statistical significance between expert and non-expert treatment. *n.s.* not significant

The comparison of the factors influencing the decision of arthroscopic procedures between expert and non-expert surgeons is shown in Table 7. Experts and non-experts named similar factors when they considered whether to remove or repair the meniscus – damage type, damage zone and patient age. However, in regard to meniscus repair qualifications, experts stated that age did not matter significantly more frequently than non-experts (*p* = 0.002).

**Table 7:** Comparison of the factors influencing the decision on arthroscopic procedures between expert and non-expert surgeons

Parameter:	All (*n* = 205)	Experts (*n* = 55)	Non-experts (*n* = 150)	*p* value
FACTORS INFLUENCING THE REMOVE/REPAIR DECISION:				
Damage type	175 (85%)	50 (91%)	125 (83%)	n.s.
Damage zone	167 (76%)	46 (84%)	121 (82%)	n.s.
Patient’s age	151 (74%)	37 (67%)	114 (76%)	n.s.
Time since injury	118 (58%)	30 (54%)	88 (59%)	n.s.
Physical activity	105 (51%)	24 (44%)	81 (54%)	n.s.
Accompanying damage	44 (23%)	14 (25%)	30 (20%)	n.s.
Damage representation in magnetic resonance	75 (36%)	22 (40%)	53 (35%)	n.s.
Sport discipline practiced by the patient	103 (50%)	23 (42%)	80 (54%)	n.s.
PATIENT’S AGE INFLUENCE ON MENISCUS REPAIR QUALIFICATION:				
Less than 50 years old	45 (22%)	10 (18%)	35 (23%)	n.s.
Less than 40 years old	34 (16%)	10 (18%)	24 (16%)	n.s.
Less than 60 years old	23 (11%)	2 (4%)	21 (14%)	0.009
Age does not matter	86 (42%)	31 (56%)	55 (36%)	0.002

*p* value is presented to establish statistical significance between expert and non-expert treatment. *n.s.* not significant

At the end of the survey, the participating orthopaedists were asked to choose the recommended procedure for traumatic meniscus tears in 18-year-old and 30-year-old professional football players. A total of 179 (87%) orthopaedists decided to repair the damaged part of the meniscus in an 18-year-old patient: 53 (97%) experts and 126 (84%) non-experts. A total of 166 (81%) surgeons decided to repair the damaged part of the meniscus in a 30-year-old patient: 44 (80%) experts and 122 (81%) non-experts.

## Discussion

The most important finding of the present study was the agreement between expert and non-expert arthroscopic knee surgeons in most aspects of clinical care. This survey explored numerous aspects of the perioperative and postoperative care of patients undergoing knee arthroscopy.

A consensus among Polish orthopaedists was reached in the preferential use of regional anaesthesia for knee arthroscopy. This is in agreement with world standards [20–23]. Regional anaesthesia, in contrast to general anaesthesia, is a simple, safe technique that is well accepted by patients and reduces the length of hospital stay. Therefore, experts and non-experts agreed on the short duration of hospital stay after knee arthroscopy (1–2 days). Polish surgeons also agreed on the lack of need for the routine recommendation of using a knee orthosis, which is in agreement with previous studies, showing no beneficial effect of bracing after knee arthroscopy [24, 25] or even indirect prevention of ACL re-rupture in cases of rehabilitation without a knee brace [26].

Pain control after knee arthroscopy is an important aspect of the patient experience. In this survey, all surgeons agreed that there is no need for intraarticular knee medications immediately after knee arthroscopy. This did not differ between the expert and non-expert surgeons. The presentation of pain is determined by the procedure of knee surgery, and previous studies have shown that a significant proportion of patients have only very mild or mild pain after knee arthroscopic procedures [27].

The current guidelines for thromboprophylaxis recommend the use of vitamin K antagonists (e.g., warfarin), low-molecular-weight heparins (LMW heparin) or aspirin [28–30]. Polish experts and non-experts agreed on the use of LMW heparin, following the recommendations regarding venous thromboembolism prevention in orthopaedic surgery and traumatology developed by Polish orthopaedic surgery experts under the auspices of the National Consultant for Orthopaedic Surgery and Traumatology and the chairman of the Polish Society for Orthopaedic Surgery and Traumatology [31].

Postoperative rehabilitation is crucial to achieve successful outcomes in patients undergoing knee arthroscopy [32], and the role of the surgeon is to educate patients about its importance. Polish surgeons agreed that proper postoperative rehabilitation of the knee is essential for returning to an active lifestyle. In our survey, 99% of the surgeons reported that they discussed the importance of compliance with the rehabilitation protocol with the patient. However, there is still room for improvement, since 1% of surgeons never recommend rehabilitation, 5% - rarely and 7% - only sometimes. In contrast to non-experts, experts admitted that their patients comply with the rehabilitation protocol to a high extent. This might be explained by the greater authority of more experienced surgeons. Polish experts recommended starting rehabilitation on the day of surgery. Surgeons from all over the world have increasingly emphasized early mobilization, which may produce favourable postoperative outcomes [33–35]. Most surgeons (92%) reported that they always recommended that their patient undergo rehabilitation with a physiotherapist after knee arthroscopy, which is now considered the gold standard, and its effectiveness has been shown by a number of control studies [36–39]. Expert surgeons did not use physical therapists as much as non-experts in making a decision regarding returning to activity. This may be due to newer surgeons being more conservative, relying on physical therapists for another opinion. Evidence-based medicine (EBM) does not exist in physical therapy, in contrast to physiotherapy. In this survey, experts and non-experts recommended physical therapy less frequently (65%) than rehabilitation with a physiotherapist (92%). More research is needed, and consensus should be determined by the Polish National Health Fund in terms of the recommendations of physical therapy after knee arthroscopy.

There is a lack of consensus regarding the optimal postoperative protocol following meniscus repair [32]. Diverse treatment methods require individual and various rehabilitation approaches, which is why direct cooperation between the physiotherapist and the patient is so important [40]. Only 42% of Polish surgeons recommend standardized rehabilitation, and 86% confirm the dependence of the rehabilitation programme on the performed procedure. Additional studies are needed to better clarify the interplay among the tear type, repair method and optimal rehabilitation protocol.

Magnetic resonance imaging (MRI) is considered to be the most accurate method for imaging the internal knee joint structure, with sensitivities in detecting medial meniscus lesions ranging from 83 to 94% [41–43]. The ESSKA meniscus consensus group recommended MRI when arthroscopy is considered to identify concomitant pathologies [17]. Magnetic resonance as a diagnostic test for meniscus damage was recommended by 97% of orthopaedists in this study. However, 50% of surgeons recommended ultrasound as a diagnostic method, which should not be practised according to the ESSKA meniscus consensus for traumatic or degenerative damage. Experts and non-experts should be educated on this.

Surgical expertise was significantly associated with the performance of the reconstructive procedures in comparison to diagnostic arthroscopy, which was performed more often by non-experts. Experts were significantly more likely to perform meniscus suture procedures than non-experts, as these procedures are considered advanced and challenging techniques. The clinical experience of participating in this survey of orthopaedists was correlated with the use of newly established methods. Experts were deciding to use bone marrow cells, biomaterials or autologous adipose tissue as meniscus repair methods. All of these approaches that involve the use of cells and biomaterial scaffolds have recently gained increasing attention as potential regenerative therapies in the field of musculoskeletal medicine [4]. Therefore, the observation that non-experts are less frequently choosing these options could be explained by their limited experience with new therapeutic options for patients, as they are still gaining experience with traditional meniscus treatment methods.

Non-expert surgeons were less likely to use objective physical tests, recommended a longer period from meniscus suture to full-range knee motion and admitted that patient age does matter for meniscus repair qualification. All of these issues might be correlated with less experience.

Both experts and non-experts preferred to suture traumatic meniscus tears in 18-year-old and 30-year-old football players. This proves the willingness of meniscus repair and awareness of its role in knee arthritis prevention.

An obvious strength of the study is that it was the first such developed survey study among Polish Arthroscopy Society members. This study had limitations. The questionnaire included 35 questions, which is a prominent number and could cause potential weariness and careless or ill-considered answers. However, during the pilot study, the average time for completion did not exceed 10 min, and it would be difficult to collect detailed information about the postoperative aspects of care with fewer questions. Defining the level of expertise at a cut-off level of more than 100 arthroscopies performed per year could be considered a biased decision for this study. Further studies are required to demonstrate clinical comparisons or second-look arthroscopy outcomes.

## Conclusions

The present survey provided useful recommendations for clinical decision-making regarding the management of knee arthroscopy. Agreement was found among almost all issues of meniscus tear treatment between experts and non-experts; however, the rehabilitation issues differed in both groups. Surgical expertise was associated with the performance of advanced meniscus repair techniques.

## Supplementary information

**Additional file 1.** A survey on orthopedists opinion for an arthroscopic treatment of meniscus injuries. A survey which was presented to orthopaedists. The questionnaire contains 35 questions regarding general arthroscopy and postoperative management

## Data Availability

The datasets used during the current study are available from the corresponding author on reasonable request.

## Abbreviations

AAT: Autologous adipose tissue
BMC: Bone marrow cells
EBM: Evidence Based Medicine
ESSKA: European Society for Sports Traumatology, Knee Surgery and Arthroscopy
LMW heparin: Low-molecular-weight heparine
MRI: Magnetic resonance imaging
PRP: Platelet rich plasma
