# Supplementary material for: Clinical practice and postoperative rehabilitation after knee arthroscopy vary according to surgeons’ expertise: a survey among polish arthroscopy society members
Source: BMC Musculoskelet Disord. 2020 Sep 23;21:626. doi: 10.1186/s12891-020-03649-9 (PMC7513306; doi:10.1186/s12891-020-03649-9)
Supplement: Supplementary file 1 — Additional file 1. A survey on orthopedists opinion for an arthroscopic treatment of meniscus injuries. A survey which was presented to orthopaedists. The questionnaire contains 35 questions regarding general arthroscopy and postoperative management [file 12891_2020_3649_MOESM1_ESM.pdf]

A survey on orthopedists opinion for an arthroscopic treatment of meniscus injuries

A. Physician's level of education and experience

a. **How many knee arthroscopies did you participate as an assistant during your residency / specialization?**

☐ 0 ☐ 1-10 ☐ 10-30 ☐ > 30

b. **How many knee arthroscopies do you perform yourself per year?**

☐ 0-10 ☐ 10-30 ☐ 30 – 50 ☐ 50-100 ☐ 100-200 ☐ 200-500 ☐ >500

c. **How many knee arthroscopies did you perform yourself during career?**

☐ 0-10 ☐ 10-30 ☐ 30 – 100 ☐ 100-500 ☐ 500-1000 ☐ 1000-2000
☐ >2000

d. **Arthroscopy of which joints you are currently performing?**

☐ shoulder ☐ elbow ☐ wrist ☐ spine ☐ hip ☐ knee ☐ ankle

B. Anesthesia for surgery

a. **What type of anesthesia do you usually administer to the patient to perform knee arthroscopy?**

☐ general anesthesia

☐ regional anesthesia (spinal and/or epidural)

☐ combination of general and regional

C. Postoperative treatment

a. **Do you always apply an orthosis to the patient?**

☐ yes ☐ no

If yes, please specify the type or brand…………………………………………………….

b. Do you use a knee drain?

☐ yes ☐ no ☐ sometimes

c. Do you use knee medications immediately after arthroscopy?

☐ yes ☐ no

If yes, please specify……………………………………………………………………

D. <u>Hospital stay</u>

a. How long does your patient usually spend in the hospital after non- reconstructive arthroscopy?

☐ hours ☐ 1 day ☐ 2 days ☐3 days ☐ 4 or more days

b. How long does your patient usually spend in the hospital after reconstructive arthroscopy?

☐ hours ☐ 1 day ☐ 2 days ☐3 days ☐ 4 or more days

c. What type of anti-thrombotic prophylaxis is the patient administered?

☐ aspirin
☐ heparin
☐ low molecular weight heparin
☐ other (please specify) ……………………….
☐ none

E. <u>Post-surgical rehabilitation</u>

a. Do you recommend your patients rehabilitation (excluding physical therapy) after knee arthroscopy?

☐ never ☐ rarely ☐ sometimes ☐ most of the times ☐ always
☐ depends on the procedure

b. How often your patients do not follow the rehabilitation recommendations?

☐ never ☐ rarely ☐ sometimes ☐ most of the times ☐ always

c. Do you talk to the patient about the need for postoperative rehabilitation?

☐ never ☐ rarely ☐ sometimes ☐ most of the times ☐ always

d. When does the rehabilitation phase for your patient officially begin?

☐ day of surgery ☐ day after surgery ☐ 2 days after surgery
☐ 3d-1 week after surgery ☐ after the first week ☐ after the first 2 weeks

e. Do all your patients follow standardized rehabilitation guidelines after knee arthroscopy?

☐ yes ☐ no

f. Do you apply different rehabilitation protocols depending on the procedures performed?

☐ yes ☐ no

g. Do you recommend the patient after knee arthroscopy rehabilitation with a physiotherapist?

☐ yes ☐ no, I set the rehabilitation program myself

h. Do you apply cryotherapy to the operated joint?

☐ yes ☐ no

i. Do you recommend physical therapy after knee arthroscopy?

☐ yes ☐ no

If yes, please specify:

☐ laser

☐ magnetotherapy

☐ ultrasounds

☐ Solux lamps

☐ ionophoresis

☐ galvanization

☐ diadynamics

☐ TENS

☐ other (specify)………………………

j. Return to sport

➢ **Who is involved in decition?**

☐ surgeon ☐ physical therapist ☐ surgeon and physical therapist ☐ patient

➢ **What criteria do you take into account when deciding on the patient's ability to return to sport? (you can select several)**

☐ time since treatment ☐ no discomfort ☐ functional state

☐ correct image in examination

k. Do you apply any objective physical test or other method in order to assess whether the patient is ready to return to sports?

☐ yes ☐ no

If yes, please specify:

☐ testing muscle strength and endurance on a dynamometer

☐ functional tests

☐ subjective surveys assessing the patient's function

☐ other (specify)…………………………………………………………………

F. **<u>Arthroscopic procedures</u>**

a. **What kind of arthroscopic procedures do you perform on knee?**

☐diagnostic arthroscopy
☐ synovial folds removal
☐ meniscus removal
☐ meniscus sewing all inside
☐ meniscus sewing inside-out/outside-in
☐ meniscus transplant
☐ ramp lesion repair
☐ microfractures
☐ cartilage reconstruction
☐ ACL reconstruction
☐ PCL reconstruction
☐ simultaneous multi-lattice reconstruction
☐ pediatric multi-ligament reconstruction (physeal-sparing)
☐ other (specify)…………………………………

b. **What kind of arthroscopic procedures do you perform most frequently on knee?**

☐diagnostic arthroscopy
☐ synovial folds removal
☐ meniscus removal
☐ meniscus sewing all inside
☐ meniscus sewing inside-out/outside-in
☐ meniscus transplant
☐ ramp lesion repair
☐ microfractures
☐ cartilage reconstruction
☐ ACL reconstruction
☐ PCL reconstruction
☐ simultaneous multi-lattice reconstruction
☐ pediatric multi-ligament reconstruction (physeal-sparing)
☐ other (specify)…………………………………

c. **What kind of meniscus repair methods do you use in your work?**

☐ sewing all inside
☐ sewing inside-out
☐ sewing outside-in
☐ scarification (pricking)
☐ platelet rich plasma (PRP)
☐ bone marrow cells

☐ autologous adipose tissue
☐ biomaterials (collagen membranes / matrices)
☐ other (specify)……………….

d. What diagnostic tests do you use when meniscus damage is suspected?
☐ none
☐ knee X-ray
☐ knee USG
☐ knee MR
☐ other (specify)……………….

e. For how long after meniscus removal do you recommend using elbow crutches?
☐ 1-6 days ☐ 1 week ☐ 2 weeks ☐ 3 weeks ☐ 4 weeks ☐ 5 weeks
☐ 6 weeks ☐ 7 weeks ☐ 8 weeks ☐ I do not recommend using crutches
☐ other (specify)...............

f. For how long after meniscus repair do you recommend using orthosis?
☐ 1-6 days ☐ 1 week ☐ 2 weeks ☐ 3 weeks ☐ 4 weeks ☐ 5 weeks
☐ 6 weeks ☐ 7 weeks ☐ 8 weeks ☐ I do not recommend using orthosis
☐ other (specify)...............

g. How soon after meniscus sewing do you recommend a full range of knee motion??
☐ 1-6 days ☐ 1 week ☐ 2 weeks ☐ 3 weeks ☐ 4 weeks ☐ 5 weeks
☐ 6 weeks ☐ 7 weeks ☐ 8 weeks ☐ other (specify)...............

h. **What factors do you consider when deciding to remove or repair meniscus damage? (you can select several)**
☐ patient’s age ☐ time since injury ☐ level of physical activity ☐type of damage
☐ damage zone (red/red-white/white) ☐ damage representation in MR
☐ accompanying damage ☐ other (specify)...............

i. Up to what patient’s age do you decide to repair meniscus?
☐ <20 y.o. ☐ <30 y.o. ☐ <40 y.o. ☐ <50 y.o. ☐ <60 y.o. ☐ <70 y.o.
☐ patient’s age does not matter

j. Whether the decision to repair / remove the meniscus depends on the practiced sport discipline?
☐ yes ☐ no

k. What is your preferred procedure in case of meniscus damage in an 18-year-old professional football player?

☐ removal of the damaged part of the meniscus

☐ repair of the damaged part of the meniscus

l. What is your preferred procedure in case of meniscus damage in a 30-year-old professional football player?

☐ removal of the damaged part of the meniscus

☐ repair of the damaged part of the meniscus
